# A Soft Sensor to Estimate the Opening of Greenhouse Vents Based on an LSTM-RNN Neural Network

**DOI:** 10.3390/s23031250

**Published:** 2023-01-21

**Authors:** Mounir Guesbaya, Francisco García-Mañas, Francisco Rodríguez, Hassina Megherbi

**Affiliations:** 1 LI3CUB Laboratory, Department of Electrical Engineering, University of Biskra, BP 145 RP, Biskra 07000, Algeria; 2 Department of Informatics, University of Almería, CIESOL, ceiA3, E04120 Almería, Spain; 3 LARHYSS Laboratory, University of Biskra, BP 145 RP, Biskra 07000, Algeria

**Keywords:** protected agriculture, greenhouse ventilation, machine learning, long short-term memory, virtual sensor, climate modeling

## Abstract

In greenhouses, sensors are needed to measure the variables of interest. They help farmers and allow automatic controllers to determine control actions to regulate the environmental conditions that favor crop growth. This paper focuses on the problem of the lack of monitoring and control systems in traditional Mediterranean greenhouses. In such greenhouses, most farmers manually operate the opening of the vents to regulate the temperature during the daytime. Therefore, the state of vent opening is not recorded because control systems are not usually installed due to economic reasons. The solution presented in this paper consists of developing a Long Short-Term Memory Recurrent Neural Network (LSTM-RNN) as a soft sensor to estimate vent opening using the measurements of different inside and outside greenhouse climate variables as input data. A dataset from a traditional greenhouse located in Almería (Spain) was used. The data were processed and analyzed to study the relationships between the measured climate variables and the state of vent opening, both statistically (using correlation coefficients) and graphically (with regression analysis). The dataset (with 81 recorded days) was then used to train, validate, and test a set of candidate LSTM-based networks for the soft sensor. The results show that the developed soft sensor can estimate the actual opening of the vents with a mean absolute error of 4.45%, which encourages integrating the soft sensor as part of decision support systems for farmers and using it to calculate other essential variables, such as greenhouse ventilation rate.

## 1. Introduction

Nowadays, agriculture faces numerous challenges, mainly, the growth of the world population, the effects of climate change, strict market regulations, and energy inefficiency [1]. Overcoming these challenges requires the application of robust and adaptive management strategies that involve the implementation of technology at all stages of the hierarchical agricultural production system. Greenhouses are prominent facilities that can help to address such challenges [2]. Greenhouses are designed to protect crops and provide suitable environmental conditions to favor their growth. The climate inside a greenhouse can be regulated using different systems such as ventilation, heating, humidification, and CO_2_ injection, among others. These actuators are necessary to regulate essential variables like air temperature, humidity, or CO_2_ concentration to improve the growth of plants and fruits [3]. This requires sensors that measure the evolution of the variables of interest to provide farmers and automatic controllers with the information needed to determine when to activate greenhouse actuators to improve crop conditions or protect them.

Traditionally, sensors and automatic controllers are not installed in greenhouses due to the associated costs. Farmers are used to manually activating the actuators based on their own experience and continuous supervision of the crop and the weather. In recent years, with the availability of low-cost devices, monitoring and control systems are more common in commercial greenhouses, mainly due to the proliferation of emerging technologies such as wireless sensor networks (WSN) [4] and the Internet of Things (IoT) [5,6], which allow farmers to remotely monitor the state of the crop in real time through mobile apps or computing platforms [7,8]. Despite the proven advantages that monitoring and control systems can offer in terms of increased crop productivity and energy efficiency [9], it is still unusual to find a high level of technology in most traditional greenhouses [10].

In this context, this paper focuses on the problem of the lack of monitoring and control systems in traditional Mediterranean greenhouses. These greenhouses operate in warmer climatic conditions and with higher values of solar radiation. Therefore, not many actuators are typically used, as the control actions are mainly focused on air temperature regulation, which directly affects crop growth [3]. In greenhouses located in warm climate zones, natural ventilation is the most widely used system to regulate air temperature during the daytime [11] due to its low cost [12]. It consists of opening and closing the greenhouse vents up to a certain point depending on the desired air temperature for the crop, allowing an exchange of the hot air leaving the greenhouse with the cooler air entering it. Most farmers manually operate the opening of the vents in these greenhouses, which means that it is not regulated by a controller. For that reason, the state of vent opening is not usually recorded, although potentiometers could be used for this purpose [13]. In this regard, it is important to note that some farmers use monitoring devices, such as commercial meteorological stations. However, these stations are equipped and designed with a limited number of sensors that only measure the evolution of the main climate variables [14]. Therefore, even with such monitoring systems, the state of vent opening is often not recorded unless a control system is used for ventilation or specific sensors are installed in the vents.

The importance of knowing the state of vent opening (generally, the state of the actuators) lies in the possibility of performing a more detailed analysis of the climatic information measured in a greenhouse, such as for modeling tasks or climate prediction. It is essential for IoT platforms as decision support systems that provide recommendations to farmers after analyzing the data measured by the stations installed in greenhouses [8]. Such recommendations are based on predictive models of greenhouse climate and crop growth [3,15], which require as input the state of vent opening to quantify the relevant effect of the ventilation flux (airflow rate) on the inside climate and crop [16,17]. In addition, continuous recording of the state of vent opening could also be important for legal aspects. For example, greenhouse structures can be damaged on stormy days by heavy wind and rain, and the recorded information on vent opening may be necessary for insurance claims.

The solution proposed in this paper consists of developing a soft sensor to estimate the state of vent opening when it is not measured in a greenhouse. Soft sensors, also known as virtual sensors, are useful tools for estimating a variable from the measurements provided by other physical sensors installed in a given system [18]. There are numerous examples of soft sensors applied to different fields, and in particular, some studies were applied to greenhouses, such as to design irrigation controllers or estimate the leaf area index of the crop [19,20,21]. Soft sensors can be classified into two categories: model-based and data-based. Model-based soft sensors use mathematical expressions that represent the relationship between the variable to be estimated and the other variables that are measured with physical sensors. For data-based soft sensors, the relationships between the estimated and measured variables can be determined using identification techniques and statistical or machine learning (ML) methods [22].

To the best of our knowledge, no previous work has been published on the estimation of the vent opening of greenhouses because it is assumed to be a measurable variable. This work is the first attempt to implement a tool for this purpose. In this sense, there are no mathematical models that directly relate the greenhouse climate to the opening of the vents. Instead, this relationship has been studied in the literature for calculating the ventilation flux, which is the airflow that circulates through the vents when they are open [16,23]. There are some well-known models for the calculation of the ventilation flux [24,25,26] that use mathematical expressions fed with the state of vent opening measured by physical sensors installed in the vents (e.g., potentiometers). These models consist of nonlinear equations with parameters that must be calibrated for different types of greenhouses. Therefore, using these models to develop a soft sensor for vent opening would be complicated. If the equations of the cited models are inverted, the vent opening could be estimated, but then actual measurements of the ventilation flux would be obligatory, which would require installing expensive sensors, such as sonic anemometers [27].

For the reasons explained above, this work aims to develop a data-driven soft sensor to estimate vent opening. To design the soft sensor, Deep Learning (DL) methods [28] are known to be a suitable option due to the satisfactory results presented in other applications for greenhouses [29]. In particular, neural networks based on Long Short-Term Memory (LSTM) are of particular interest for the described problem, considering the complexity and nonlinearity of the dynamics involved in greenhouses [30]. Consequently, an LSTM-based network has been selected for the implementation of the soft sensor due to its powerful advantages and ability to deal with the vanishing gradient problem. This capability is expected to be advantageous for successfully modeling some delayed and correlated dynamics of the greenhouse inside climate [31].

**Figure 1:**
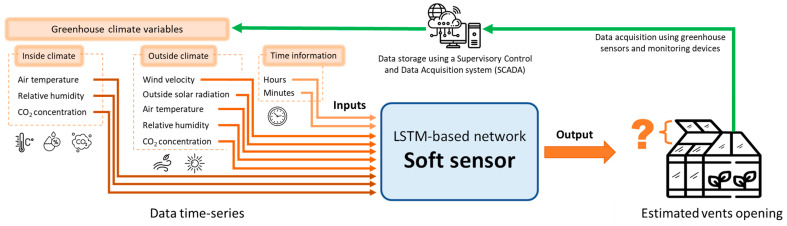
Conceptualization of the soft sensor to estimate the opening of greenhouse vents.

In summary, the main contribution of this work is the development of a soft sensor using an LSTM-based network to estimate the opening of greenhouse vents from climate variables commonly measured in medium-technology greenhouses. Figure 1 presents the concept of the developed soft sensor, which receives as inputs the measurements of the following climate variables recorded inside and outside a greenhouse: air temperature, air relative humidity, global solar radiation, CO_2_ concentration in the air, and outside wind velocity. The estimation of vent opening with a data-based soft sensor is possible because the evolution of the inside climate variables is affected by the ventilation flux. Every time that the vents of a greenhouse are opened or closed, variations in all or some of the aforementioned variables are measured. A historical dataset from a traditional Mediterranean greenhouse was used to train and test a series of LSTM-based network architectures to reproduce the actual opening of the vents which caused the measured variations in the inside climate variables. The training and testing processes were performed using a progressive elimination procedure (PEP) before selecting the final soft sensor based on an LSTM-RNN neural network. The results show a satisfactory performance, demonstrating that the developed soft sensor can estimate the actual opening of the vents with a reduced error.

The soft sensor’s contribution lies in providing an estimated signal of the opening of vents for its possible use by the existing models to calculate the ventilation flux and predict greenhouse climate variables [23,24]. In this sense, the soft sensor would allow the calculation of the ventilation flux when no physical sensors are available in a greenhouse to measure the opening of the vents. Calculating the ventilation flux is important for the predictive modeling of other greenhouse climate variables that are strongly affected by it, such as relative humidity, CO_2_ concentration, or air temperature. Other potential applications of the soft sensor may include its integration into IoT platforms, as discussed above, which would be useful for continuous estimation of vent opening from data measured with commercial weather stations installed in greenhouses.

The remainder of the paper is organized as follows. In Section 2, materials and methods are described. In Section 3, the results of training and testing the soft sensor using an LSTM-based network are presented and discussed. Finally, the conclusions of the work are summarized in Section 4.

## 2. Materials and Methods

In this section, an experimental greenhouse was used to obtain the dataset needed for this work. The data obtained include a set of inside and outside greenhouse climate variables to be analyzed and selectively used as inputs in the next section, as well as the target, which is the actual vent-opening signal generated by an automatic controller. As a result of changes in the opening of vents, the climate variables inside the greenhouse are affected by the ventilation flux. Thus, the theory of this work is that a soft sensor based on an LSTM neural network can estimate the vent opening that causes those changes in the inside climate variables. The components and equations that constitute the LSTM-based neural network are explained in this section. Furthermore, the potential network architectures and their hyperparameters are preselected.

### 2.1. Greenhouse Description

**Figure 2:**
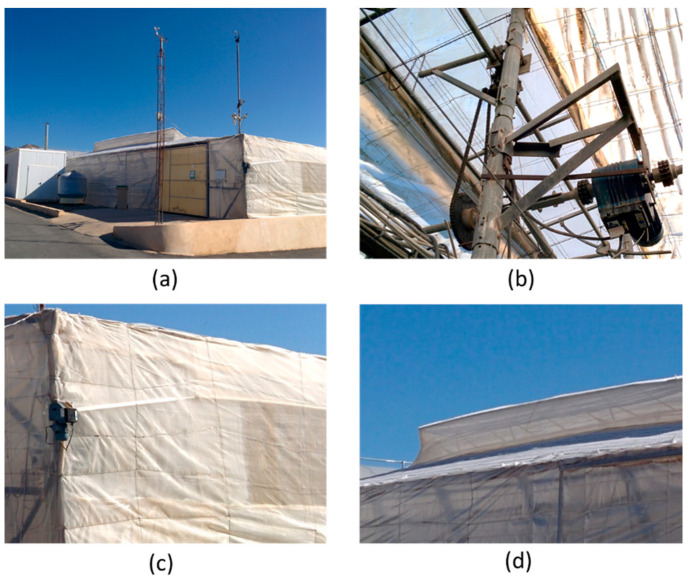
Experimental greenhouse: (**a**) Outside view; (**b**) Detail of a motor to open the roof vents; (**c**) Detail of a lateral vent with its motor to roll up the plastic; (**d**) Detail of a roof vent.

The greenhouse used in this study is located at “Las Palmerillas” Experimental Station of the Cajamar Foundation in Almería, Spain, at an altitude of 151 m. It is a traditional Mediterranean greenhouse (see Figure 2a) with a surface of 877 m^2^ (37.80 m × 23.20 m). Tomato (*Lycopersicon esculentum* “Ramy”) is the crop grown inside this experimental greenhouse, with a plant density of 1.4 plants/m^2^. The greenhouse is equipped with auxiliary systems, such as different actuators, to control the indoor climate. Particularly, the greenhouse has five roof vents (8.36 m × 0.73 m) and two lateral vents (32.75 m × 1.90 m) for natural ventilation, situated on the north and south sides. The roof vents have an angled opening, as shown in Figure 2d, while the lateral vents are opened by rolling up a plastic film, as presented in Figure 2c. All vents can be opened from 0 to 100% of their ventilation area with a resolution of 10% by means of three electric motors (see Figure 2b), which can be manually or automatically operated.

### 2.2. Experimental Dataset

A dataset with 81 recorded days (233,280 samples) was used to train, validate, and test the developed soft sensor. This dataset was acquired using a commercial data acquisition system called Compact FieldPoint (National Instruments, Austin, TX, USA). It contains 13 climate variables related to inside and outside air temperature, relative humidity, global solar radiation, CO_2_ concentration in the air, wind velocity, vent opening, and time variables. The data were recorded during the growth cycle of a tomato crop using sensors installed inside and outside the experimental greenhouse (see Table 1). Their acronyms and units are presented in Table 2. To capture the rapid climate changes inside the greenhouse due to the effect caused by the opening of the vents, a sampling time of 30 s was selected for the data. The opening signal of vents was generated by a supervisory and control data acquisition (SCADA) system, in which a controller was executed to regulate the air temperature inside the greenhouse by natural ventilation.

**Figure 3:**
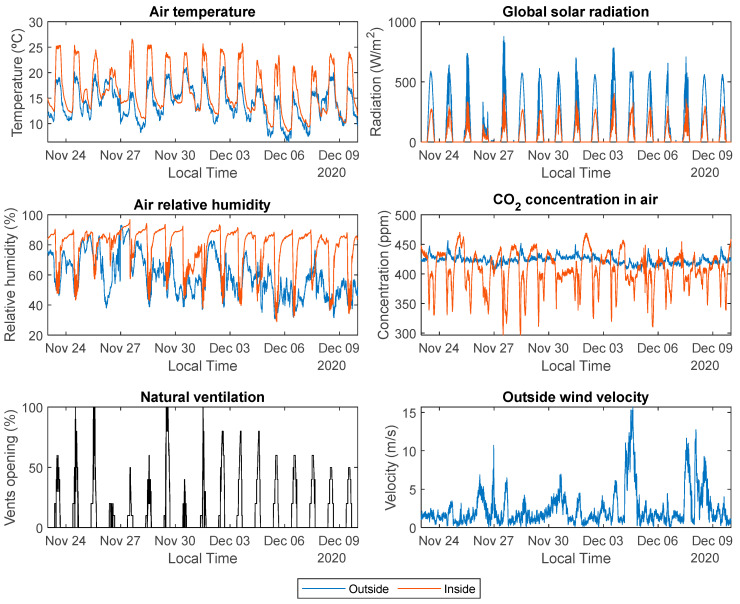
Example of some days contained in the experimental dataset from 10 October 2020 to 29 December 2020.

The period for the selected dataset was from 10 October to 29 December 2020. Due to the large size of this dataset, Figure 3 shows an example of the data recorded between 24 November and 9 December 2020. Notice that the selected data represent the usual dynamics in the greenhouse, with a mix of sunny, cloudy, and windy days. The opening signal of the vents presents different amplitudes and changes due to the action of the automatic control system, and also some days with a less variant behavior, similar to the manual operation performed by farmers, as can be observed from 3 to 9 December 2020.

### 2.3. Long Short-Term Memory

In artificial neural networks, the LSTM cell is a powerful deep recurrent neural system developed specifically to deal with the vanishing gradient problems that often occur when learning long-term relationships between system inputs and target outputs [32]. This fact motivates the application of LSTM-based neural networks for greenhouse climate modeling, which involves short- and long-term dependencies for multiple inputs (e.g., different climate variables) and the idea that their numerical effects would gradually vanish over time during the training of a neural network if LSTM structures were not used. For this reason, the opening of greenhouse vents could be better estimated not only on the basis of the current states of the measured climate variables but also on the basis of stored information of the long-term past states. In this sense, as a recurrent network, the output of an LSTM cell is fed back as input, creating a recursive flow of information with increased capability for information storage.

**Figure 4:**
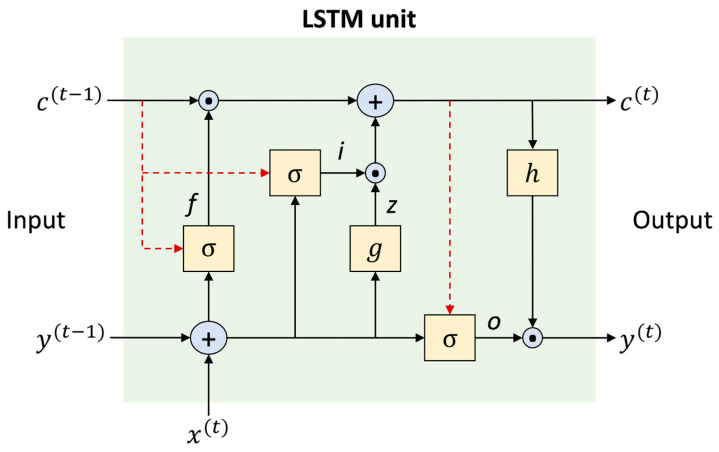
The architecture of a typical LSTM block [31].

An LSTM unit consists of four main components: a cell, an input gate, an output gate, and a forget gate. The cell remembers values over varying time intervals, and the cell gates control the flow of information. The LSTM structure consists of memory blocks, which are recurrently connected subnetworks. The memory block objective is to maintain its state over time while regulating information flow by means of nonlinear gate units. Figure 4 shows the architecture of an LSTM cell, involving an input signal x(t), an output signal y(t), the cell state $c^{\left(t\right)}$, and different activation functions σ, g, and h. The components and the way in which an LSTM block processes the flow of information are briefly explained below [31]:

Block input, z. It incorporates the current input $x^{\left(t\right)}$ and the previous value of the output $y^{\left(t-1\right)}$ of one LSTM unit. It is calculated as follows:

(1) $$z^{\left(t\right)}=g\left(W_{z\;}x^{\left(t\right)\;}+R_{z}\;y^{\left(t-1\right)\;}+b_{z\;}\right)$$

where *t* is the time instant, $W_{z}$ and $R_{z}$ are the weights for $x^{\left(t\right)}$ and $y^{\left(t-1\right)}$, respectively, and $b_{z}$ represents a bias weight vector.

Input gate, i. It is updated by merging $x^{\left(t\right)}$, $y^{\left(t-1\right)}$ and $c^{\left(t-1\right)}$ as follows:

(2) $$i^{\left(t\right)}=\sigma \;\left(W_{i}\;x^{\left(t\right)}+R_{i}\;y^{\left(t-1\right)}+p_{i}⊙\;c^{\left(t-1\right)\;}+b_{i}\right)$$

where ⊙ is a point-wise multiplication of the wights $W_{i}$, $R_{i}$, and $p_{i},$ with $x^{\left(t\right)}$, $y^{\left(t-1\right)}$, and $c^{\left(t-1\right)}$, respectively, in which $b_{i}$ is the bias vector associated with the input gate.

Forget gate, f. In this component, the LSTM unit decides which information from its previous cell states $c^{\left(t-1\right)}$ should be removed. Hence, the activation value of the forget gate at t is calculated with the following expression:

(3) $$f^{\left(t\right)\;}=\sigma \;\left(W_{f\;}\;x^{\left(t\right)}+R_{f\;}\;y^{\left(t-1\right)\;}+p_{f\;}⊙\;c^{\left(t-1\right)\;}+b_{f}\right)$$

where $W_{f}$, $R_{f}$, and $p_{f}$ are the weights associated with $x^{\left(t\right)}$, $y^{\left(t-1\right)}$, and $c^{\left(t-1\right)}$, respectively, and $b_{f}$ is the bias of the forget gate.

Cell state, c. It is calculated by merging the previous value of the cell state $c^{\left(t-1\right)}$ with the block input $z^{\left(t\right)},$ the input gate $i^{\left(t\right)}$, and the forget gate $f^{\left(t\right)}$ values as follows:

(4) $$c^{\left(t\right)}=z^{\left(t\right)}⊙\;i{\;}^{\left(t\right)}+c^{\left(t-1\right)}⊙\;f^{\left(t\right)}$$

Output gate, o. Its value is calculated with the following expression:

(5) $$o^{\left(t\right)}=\sigma \;\left(W_{o\;}x^{\left(t\right)}+R_{o\;}y^{\left(t-1\right)\;}+p_{o\;}⊙\;c^{\left(t\right)}+b_{o}\right)$$

where $W_{o}$, $R_{o}$ and $p_{o}$ are the weights associated with $x^{\left(t\right)}$, $y^{\left(t-1\right)}$ and $c^{\left(t-1\right)}$, respectively, and $b_{o}$ is a bias weight vector.

Block output, y. Finally, the block output is calculated as:

(6) $$y^{\left(t\right)\;}=h\left(c^{\left(t\right)}\right)\;⊙\;o^{\left(t\right)}$$

In Equations (1)–(6), σ, g, and h refer to the point-wise nonlinear activation functions. The function used for gate activation is the logistic sigmoid, as presented in Equation (7).

(7) $$\sigma \;\left(x\right)=\frac{1}{1+e^{1-x}}$$

The hyperbolic tangent is often used as the block input and output activation functions, g(x)=h(x)=tanh(x).

Generally, the vanishing gradient problems can be overcome by using a constant error carousel (CEC), which preserves the error signal within each cell. In a neural network, the role of the LSTM cells is to abstract a meaningful representation of the input time series and then transmit them to the additional hidden layers. Although LSTM-based networks are already performing very well, the potential for improvements is still being explored, as indicated in the comprehensive state-of-art in [31].

### 2.4. Network Architecture and Hyperparameters Preselection

The LSTM-based network employs full gradient training to adapt the learnable network parameters (weights). The Backpropagation Through Time (BPTT) technique is used to calculate the weights that connect the network components. The LSTM-based network has a set of parameters, which are called hyperparameters. They are specifically determined to define the network architecture and control the learning process in the training phase before applying it to a dataset. These hyperparameters were selected as follows:

- The number of hidden layers. It is selected by trial and error between 4 or 5 layers. Three types of hidden layers constituted the initial network architectures that were tested: LSTM cells, Dense (feedforward ANN), and RNN layers, as shown in Table 3. The reader is encouraged to find more details about RNNs and their relationship with LSTM cells in [30].
- The number of neurons. The number of network weights, which depends on the number and type of the hidden layers and the number of their neurons, is recommended to be much smaller than the number of data samples to avoid overfitting the network to the training data and to favor the generalization of the network output [33]. Hence, the number of neurons was selected accordingly, as presented in Table 3. The number of network weights remains around 18,000, which is much smaller than the number of training data samples (186,705 samples) multiplied by the number of selected inputs (8–13 inputs).
- Historical input data. By trial and error, 40 samples (20 min) were chosen as historical input data to capture all the delayed dynamics of the greenhouse climate, knowing that it presents some slow responses to disturbances (i.e., external weather conditions) and control actions as time-dependent events. It is a fundamental feature of RNNs, specifically of LTSM-based networks, which allows the selective and meaningful mapping of historical input data to the final output.
- Activation function. As presented in Equation (7), sigmoid is the selected function for all the regular layers. It is proven to be significantly useful in the multinomial logistic regression method, which can model types where the discrete output can have more than two possible discrete outcomes [34]. This is particularly important considering that the vent opening is normally a signal restricted to 11 states as discrete values ranging from 0 to 100% with 10% jumps. These jumps are due to the resolution of the motors used to open the vents in greenhouses.
- Optimizer. Adam is the selected optimizer. It is used as a mini-batch gradient descent method. It is based on adaptive estimation of first- and second-order moments. It is computationally efficient, requires little memory, and is suitable for problems with noisy and sparse gradients [35].
- Learning rate. The default learning rate of 0.001 is used for Adam. Higher and lower values were tested, but 0.001 proved to be more efficient in terms of loss reduction and computation time.
- Batch size. The batch size defines the number of samples to work within one iteration before updating the internal weights of the network. By trial and error, the batch size was set as 32 samples to accelerate the training process of the network.
- The number of epochs. An epoch is when all data samples pass through the neural network. By trial and error, 150 epochs were deemed sufficient for this study. In addition, the early stop feature is used to automatically stop the training of the network if no improvement in the validation loss function is shown for more than 50 epochs. In this case, the network with the best weights until that moment is stored. Also, the training can be stopped manually when overfitting is graphically noticed, knowing that the best network is automatically saved after every epoch.

**Figure 5:**
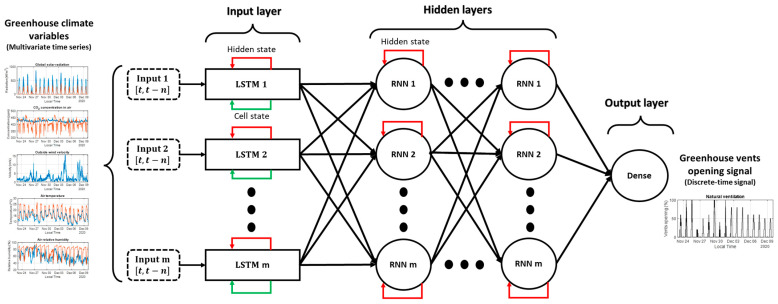
The architecture of the developed LSTM-RNN network.

In summary, the network architecture obtained in this work is presented in Figure 5.

## 3. Results and Discussion

**Figure 6:**
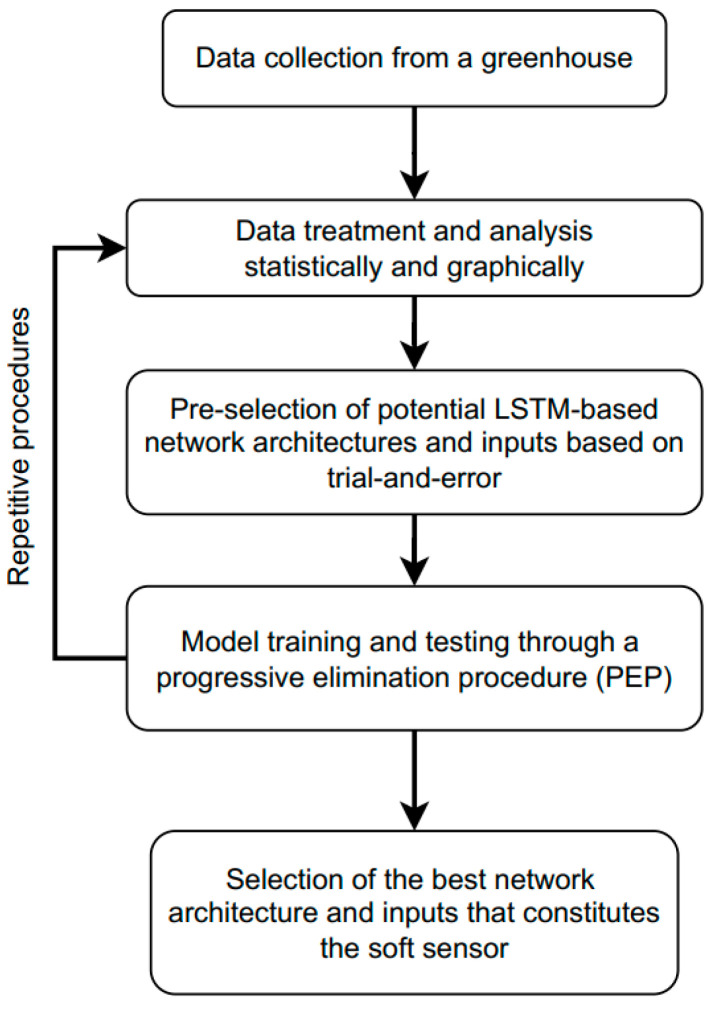
Soft sensor development methodology.

In this section, a data analysis is performed to preselect network inputs. The preselected inputs are then used to test two possible LSTM-based network architectures using supervised learning. Finally, a network architecture is selected based on statistical and graphical results and using different sets of the selected inputs in a PEP procedure. The methodology to develop the soft sensor is summarized in Figure 6. The development stages of the soft sensor were carried out using the machine learning platform called Tensorflow. The statistical evaluations are based on four loss functions which are the coefficient of determination $(R^{2}$), the mean absolute error (MAE), the maximum absolute error (MaxAE), and the root mean absolute error (RMAE). The loss function used in the training process is the mean square error (MSE). For the different tests, the computational unit used was a computer with an AMD Ryzen 5 3400G and Radeon Vega Graphics, eight cores, 3.7 GHz, and 8 GB RAM DDR4 1333 MHz. The developed soft sensor was coded and tested in Python 3.9 using the Anaconda software and Visual Studio Code editor.

### 3.1. Data Analysis and Inputs Preselection

The available data were standardized and analyzed to study the relationships between the greenhouse climate variables and the vent opening signal for the preselection of the network inputs. The analysis was carried out in two phases: statistically, using different correlation coefficients, and graphically, using regression analysis.

**Figure 7:**
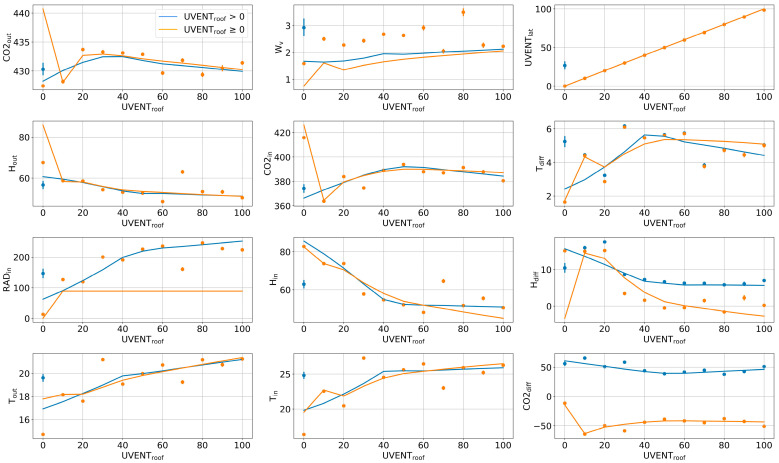
Regression analysis of the greenhouse climate variables with the vent opening in two cases: using the complete variable of vent opening signal ${\mathrm{UVENT}}_{\mathrm{roof}}\geq \;0\%$ (orange) and using only time intervals when vents are open ${\mathrm{UVENT}}_{\mathrm{roof}}>\;0\%$ (blue).

The dataset includes the opening signals for roof vents and lateral vents (${\mathrm{UVENT}}_{\mathrm{roof}}$ and ${\mathrm{UVENT}}_{\mathrm{lat}}$), which are almost identical (see their linear regression analysis in the upper right of Figure 7). In this sense, only the opening signal of the roof vents was used as the target to be estimated for simplicity. To extract additional information from the dataset and reduce the computational time of the training process, three variables $T_{\mathrm{diff}\;}$, $H_{\mathrm{diff}\;}$, and $\mathrm{CO}2_{\mathrm{diff}}$, representing differences between the inside and outside greenhouse environments, were added to the dataset as potential inputs after calculating them as follows:

(8) $$T_{\mathrm{diff}\;}={\;T}_{\mathrm{in}}-{\;T}_{\mathrm{out}\;}$$

(9) $$H_{\mathrm{diff}\;}={\;H}_{\mathrm{in}\;}-{\;H}_{\mathrm{out}\;}$$

(10) $$\mathrm{CO}2_{\mathrm{diff}\;}=\;\mathrm{CO}2_{\mathrm{in}\;}-\;\mathrm{CO}2_{\mathrm{out}\;}$$

In the statistical analysis, two cases were studied. First, using the complete signal of the vent opening (${\mathrm{UVENT}}_{\mathrm{roof}}\geq \;0\%$), and second, using only the time intervals when the vents were open (${\mathrm{UVENT}}_{\mathrm{roof}}>0\%$), as presented in Table 4. In both cases, three correlation coefficients were used: Pearson’s coefficient for the linear correlation analysis, and Spearman’s and Kendall’s rank coefficients [36] for the analysis of linear and nonlinear relationships.

As for the graphical analysis, all the data variables were graphically represented, as previously shown in Figure 3. Normally, it is expected that the vents are closed mostly at night, so the inside solar radiation measurements could be useful as an indication of daytime and nighttime periods. In addition, regression analysis to study the linear/nonlinear and monotonic/non-monotonic relationships in the data was also performed for two cases (${\mathrm{UVENT}}_{\mathrm{roof}}\geq \;0\%$ and ${\mathrm{UVENT}}_{\mathrm{roof}}>0\%$), as shown in Figure 7. The presented curves were obtained using the “regplot” function of a Python data visualization library called Seaborn [37]. This function has been developed as a practical tool to graphically demonstrate linear and nonlinear data relationships and obtain the best-fit curve. It has a useful feature called “x_estimator” for regression analysis when discrete variables are involved. It can calculate and plot the mean of the y-axis samples corresponding to each repeatable discrete value on the x-axis (a data category), which in most cases helps to demonstrate how the best-fit curve was fitted to the data distribution.

The relationships represented by the values of correlation coefficients and the results of the regression analysis are briefly discussed, and the potential inputs are initially selected accordingly in Table 5. It is commonly known that, when manually controlling the opening of greenhouse vents, farmers usually follow a predetermined time-based schedule to know when the vents should be opened or closed and what their opening percentage should be depending on the conditions of the crop, the season, and the greenhouse geographical location. For these reasons, two time-related variables, $X_{\mathrm{hours}}$ and $X_{\mathrm{minutes}}$, were also considered as inputs to the soft sensor, which may be helpful to take into account specific changes in the vent opening and climate evolutions that repeatedly occur at a given time. In summary, based on the findings of the data analyses, 10 variables were selected as inputs to the LSTM-based network: $X_{\mathrm{hours}}$, $X_{\mathrm{minutes}}$, ${\mathrm{RAD}}_{\mathrm{in}}$, $W_{v}$, $\mathrm{CO}2_{\mathrm{in}}$, $H_{\mathrm{in}}$, $T_{\mathrm{in}}$, $T_{\mathrm{diff}}$, $H_{\mathrm{diff}}$, and $\mathrm{CO}2_{\mathrm{diff}}$.

### 3.2. Training and Testing the LSTM-Based Network

The described LSTM-based network was trained and tested using different architectures and inputs in a PEP procedure to obtain the final network for the soft sensor, knowing that the target to be estimated is the greenhouse vent opening (${\mathrm{UVENT}}_{\mathrm{roof}}$). The training process consists of identifying the network weights by minimizing a loss function (MSE) that indicates the error between the real measured signal of the vent opening and the output of the trained network (i.e., the vent opening estimated by the soft sensor).

#### 3.2.1. Dataset Splitting

**Figure 8:**
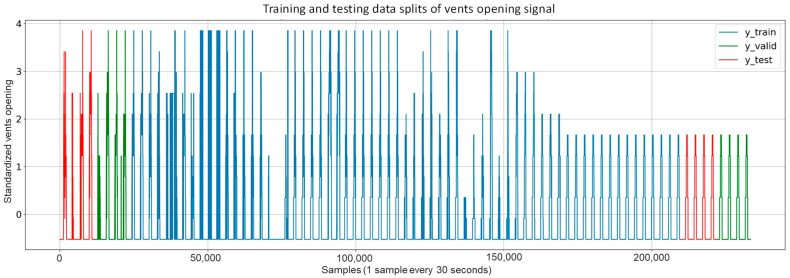
Splitting the target data into three parts: Training target (blue), Validation target (Green), and Test target (red).

The development of any ANN requires dividing the available data into three sets (for training, validation, and testing processes) manually or automatically, depending on different techniques. In this work, the time series data were manually divided based on the evolution of the target variable as one of the main factors when manually splitting data. Hence, the data were divided as presented in Figure 8, consisting of the following parts:

- A training dataset is used for the network learning process to adjust its parameters. The complete dataset includes two different control methods for the opening of the vents. One is an automatic control showing rapid changes (before sample 160,000), and the other is a time-dependent control showing fewer changes (after sample 160,000). The training dataset was selected to include both types of control for the vent opening to enhance the training process with sufficient information. Moreover, this dataset was shuffled to ensure generalization during the training process. It contains 64 days representing 80% of the total dataset, from 19 October 2020 to 22 December 2020.
- A validation dataset was also used during the training process to provide an unbiased evaluation of the network while being fitted to the training dataset. The validation dataset is also involved in other forms of network preparation, such as feature and threshold selection. The validation dataset was selected to contain 8 days representing 10% of the complete dataset: 4 days from the start of the complete dataset (from 14 October 2020 to 17 October 2020) and another 4 days from the end (from 26 December 2020 to 29 December 2020). These days were selected because they present the different types of control for the opening of the vents.
- A test dataset is used to perform an unbiased evaluation of the final network. The test dataset was also selected to contain 8 days representing 10% of the complete dataset: 4 days from the start of the dataset (from 10 October 2020 to 13 October 2020) and another 4 days from the end (from 22 December 2020 to 25 December 2020).

#### 3.2.2. Network Training and Progressive Elimination Procedure for Input Selection

The network training process was performed using different architectures and inputs. As presented in Table 3, two architectures, “A” and “B”, were preselected based on multiple tests. The first tests focused on evaluating a simple architecture (LSTM-ANN) consisting of an LSTM layer as an input layer, five hidden layers, and an output layer of a dense type. Secondly, a deep network (LSTM-RNN) was tested consisting of an LSTM layer as an input layer, four RNN hidden layers to increase the capability of the resulting network, and a dense output layer. The LSTM layer was used in both cases as an input layer to take advantage of its ability to abstract a meaningful representation of the input time series, and then the extracted higher-level information was transmitted to the hidden layers in order to produce the output, which is the estimated vent opening signal.

The preselected architectures were trained, tested, and statistically evaluated with different inputs in a PEP procedure, as presented in Table 6. According to the input preselection in Section 3.1 and the greenhouse climate dynamics, the first PEP procedure consisted of eliminating the input $W_{v}$ because it was not correlated with the opening signal of vents. It is a very noisy variable that was graphically observed to cause undesirable fluctuations in the evolution of the output of the networks (i.e., the estimated vent opening). The second PEP procedure consisted of preserving $W_{v}$ and eliminating $T_{\mathrm{out}}$, $H_{\mathrm{out}}$, and $\mathrm{CO}2_{\mathrm{out}}$ because these variables do not change when the vents are opened or closed, and their physical effects on the greenhouse climate are already taken into account in the calculated climate differences, $T_{\mathrm{diff}}$, $H_{\mathrm{diff}}$, and $\mathrm{CO}2_{\mathrm{diff}}$. It was concluded that the elimination of these inputs resulted in a decrease in the error values; thus, the PEP is an efficient procedure for improving the estimation and reducing the size of the data.

The time consumed for the training processes of the LSTM-ANN network is around 6 h, and for the LSTM-RNN network, it is around 9 h, which is considered an acceptable time consumption with a moderate computational cost. According to the results presented in Table 6, architecture “B” with an LSTM-RNN network outperforms architecture “A” with an LSTM-ANN network. Moreover, based on the PEP procedure, the best results for the LSTM-RNN network are obtained using only 10 selected inputs which are: $X_{\mathrm{hours}}$, $X_{\mathrm{minutes}}$, ${\mathrm{RAD}}_{\mathrm{in}}$, $W_{v}$, $\mathrm{CO}2_{\mathrm{in}}$, $H_{\mathrm{in}}$, $T_{\mathrm{in}}$, $T_{\mathrm{diff}}$, $H_{\mathrm{diff}}$, and $\mathrm{CO}2_{\mathrm{diff}}$.

**Figure 9:**
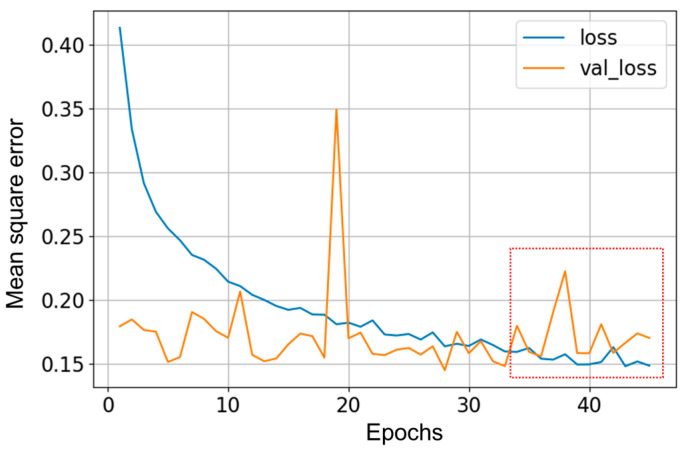
Evolution of the loss function during training (loss) and validation (val_loss) processes using the standardized data.

**Figure 10:**
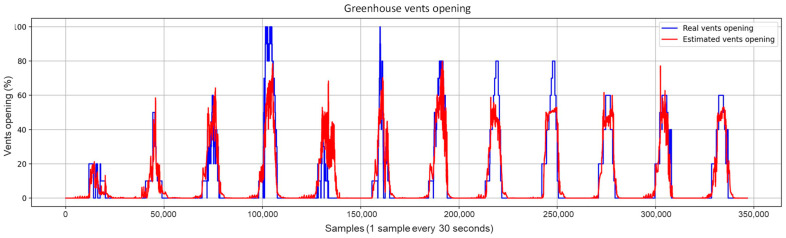
A sample from the results of the training process of the LSTM-RNN network.

**Figure 11:**
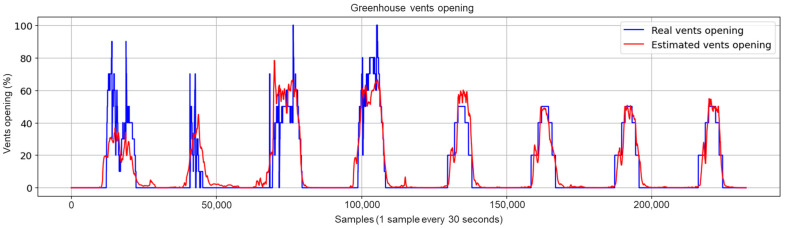
Test results for the LSTM-RNN network with the final selected 10 inputs (after eliminating $T_{\mathrm{out}}$, $H_{\mathrm{out}}$ and ${\mathrm{CO}2}_{\mathrm{out}}$).

**Figure 12:**
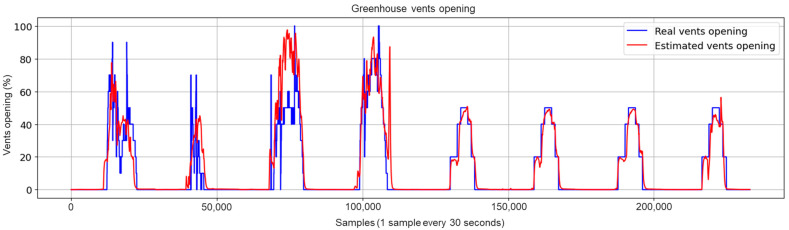
Test results for the LSTM-RNN network with 12 inputs (after eliminating $W_{v}$).

**Figure 13:**
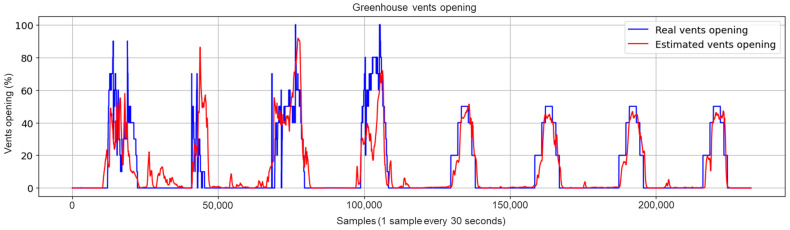
Test results for the LSTM-RNN network using all the available 13 inputs.

Concerning the graphical evaluation, the training process of the best LSTM-RNN network presented an adequate convergence for the evolution of the training and validation cost function, as shown in Figure 9. The training process was manually stopped when the onset of divergence (see the red box in Figure 9) was observed as a sign of network overfitting. An example of estimation results using the training data is shown in Figure 10, which presents a satisfactory fit between the actual and estimated vents opening, avoiding overfitting to the training data. The results using the test dataset with the 10 selected inputs are shown in Figure 11. In addition, Figure 12 and Figure 13 show other results using the test dataset with 12 inputs and 13 inputs, respectively. The estimated opening of vent results were filtered to present a less noisy signal. A first-order filter was used for the output of the LSTM-RNN network, with a time constant of 250 s. In Figure 11, the results obtained with the LSTM-RNN network show a satisfactory fit to the real vent opening by reproducing the time intervals in which the vents are opened and closed, as well as the maximum opening amplitudes, and by estimating the main changes in the signal. It can be noticed that the fit is better in one part (see samples after 12,000) than in another due to the different evolution of the opening signal of vents. It is less challenging for the network to estimate the part of the signal with fewer changes per day because it is a repetitive dynamic, and the changes in the opening values occur more slowly and far apart over time. These two factors allow the network to learn more about this part of the signal than the variant part. In other words, the fewer changes per day in the opening of the vents, the easier it is for the network to interpret the corresponding change in the greenhouse climate and the more accurate the estimation provided by the soft sensor. This fact is particularly interesting for most traditional greenhouses, in which farmers manually open and close the vents in a similar way, so these results confirm the usefulness of the developed soft sensor in that context. However, it can be concluded that it will be necessary to train the LSTM-RNN network with larger datasets to increase the accuracy of estimating the rapid changes in vent opening.

## 4. Conclusions

A soft sensor based on an LSTM-RNN neural network has been developed to estimate the opening of greenhouse vents using a set of measurable climate variables. A comprehensive statistical and graphical data study was performed using different linear and nonlinear correlation coefficients and regression analyses. Based on the results of this data analysis and trial-and-error training processes, two possible network architectures (LSTM-ANN and LSTM-RNN) and ten inputs were preselected for the soft sensor design. In addition, a series of training and testing processes were carried out in a PEP procedure. It has been shown that the external climate variables $T_{\mathrm{out}}$, $H_{\mathrm{out}}$, and $\mathrm{CO}2_{\mathrm{out}}$ are necessary to calculate the corresponding differences between the inside and outside greenhouse climate $T_{\mathrm{diff}}$, $H_{\mathrm{diff}}$, and $\mathrm{CO}2_{\mathrm{diff}}$ to be used as inputs to the LSTM-based network. It has also been found that $H_{\mathrm{diff}}$ is the most correlated input presenting a negative monotonic nonlinear correlation with the opening signal of vents.

The best network architecture is the LSTM-RNN, due to its performance in estimating the actually recorded opening of vents with reduced error values: $R^{2}=0.8,\;\mathrm{RMSE}=9.13\%,\;\mathrm{MAE}=4.45\%$ and MaxAE=62.94%. As for the graphical results, the soft sensor developed using the LSTM-RNN network provides a good fit between the estimated and the real vent opening in both daytime and nighttime. However, the estimation is more accurate when there are fewer changes in the opening of vents. Moreover, it has not been possible to compare the obtained network and results with other works since this study is the first attempt to estimate the opening of greenhouse vents.

Consequently, the results confirm that the soft sensor is suitable for use in greenhouses where farmers manually operate the opening of the vents. In this context, the soft sensor could be applied to:

- Estimate and monitor the evolution of the natural ventilation flux.
- Develop predictive models for greenhouse climate evolution as a function of the estimated vent opening.
- IoT platforms and decision support systems to provide recommendations to farmers after analyzing the measured data, and for example, alert them to close the vents whenever a high wind velocity is detected to avoid any risk of damage to the greenhouse and crop.

In conclusion, the contribution of this work to the field of greenhouse agriculture lies in the possibility of offering a tool that can be applied to estimate the opening signal of vents without the need to have installed specific sensors on the vents of a greenhouse or control systems for natural ventilation.

In future works, the use of larger datasets to improve the performance of the soft sensor will be studied. The focus will be on improving the estimation results when fast changes in the vent opening occur. The soft sensor could also be tested in different greenhouses with different shapes and geographical locations.

## Figures and Tables

**Table 1:** Sensors installed outside and inside the experimental greenhouse.

Sensor	Brand	Model	Precision	Range
Air temperature	Campbell Scientific	HC2S3	±0.1 °C	−40 to 60 °C
Air relative humidity	Campbell Scientific	HC2S3	±0.1%	0 to 100%
Global solar radiation	Hukseflux	LP02	<±1%	0 to 2000 W/m^2^
CO_2_ concentration in air	E+E Elektronik	EE820-C2	<±50 ppm	0 to 2000 ppm
Wind velocity	Vector Instruments	A100L2/PC3	<2%	0 to 75 m/s

**Table 2:** Dataset variable acronyms, descriptions, and units.

Acronyms	Description	Unit
$T_{\mathrm{in}}$	Inside air temperature	°C
$T_{\mathrm{out}}$	Outside air temperature	°C
$H_{\mathrm{in}}$	Inside air relative humidity	%
$H_{\mathrm{out}}$	Outside air relative humidity	%
$\mathrm{CO}2_{\mathrm{in}}$	Inside CO_2_ concentration	ppm
$\mathrm{CO}2_{\mathrm{out}}$	Outside CO_2_ concentration (it can be considered constant as 400 ppm)	ppm
${\mathrm{RAD}}_{\mathrm{in}}$	Inside solar radiation	W/m^2^
$W_{v}$	Outside wind velocity	m/s
$\mathrm{CO}2_{\mathrm{diff}}$	Difference between inside and outside CO_2_	ppm
$H_{\mathrm{diff}}$	Difference between inside and outside relative humidity	%
$T_{\mathrm{diff}}$	Difference between inside and outside air temperature	°C
${\mathrm{UVENT}}_{\mathrm{roof}}$	Vent opening signal	%

**Table 3:** Selection of network architecture: number and type of layers and number of neurons.

	Type of Layers		Number of Neurons	
	Architecture A (LSTM-ANN)	Architecture B (LSTM-RNN)	Architecture A (LSTM-ANN)	Architecture B (LSTM-RNN)
Input layer	LSTM	LSTM	Number of inputs	Number of inputs
First hidden layer	Dense	RNN	50	30
Second hidden layer	Dense	RNN	80	60
Third hidden layer	Dense	RNN	80	60
Fourth hidden layer	Dense	RNN	50	30
Fifth hidden layer	Dense	/	15	/
Output layer	Dense	Dense	1 neuron for 1 target	1 neuron for 1 target

**Table 4:** Statistical analysis of the linear and nonlinear correlation.

	Pearson’s Coefficient		Spearman’s Rank Coefficient		Kendall’s Rank Coefficient	
	$UVENT_{roof}$ ≥ 0%	$UVENT_{roof}$ > 0%	$UVENT_{roof}$ ≥ 0%	$UVENT_{roof}$ > 0%	$UVENT_{roof}$ ≥ 0%	$UVENT_{roof}$ > 0%
$\mathrm{CO}2_{\mathrm{out}}$	0.22	−0.05	0.09	−0.02	0.07	−0.02
$H_{\mathrm{out}}$	−0.34	−0.15	−0.03	−0.18	−0.02	−0.13
${\mathrm{RAD}}_{\mathrm{in}}$	0.75	0.37	0.07	0.45	0.05	0.34
$T_{\mathrm{out}}$	0.5	0.3	0.17	0.32	0.14	0.23
$W_{v}$	0.2	0.03	−0.01	0.11	−0.01	0.08
$\mathrm{CO}2_{\mathrm{in}}$	−0.3	0.14	−0.03	0.2	−0.02	0.14
$H_{\mathrm{in}}$	−0.07	−0.46	−0.03	−0.51	−0.02	−0.38
$T_{\mathrm{in}}$	0.6	0.32	0.08	0.32	0.07	0.24
$\mathrm{CO}2_{\mathrm{diff}\;}$	−0.36	0.14	0.1	−0.19	0.07	−0.13
$H_{\mathrm{diff}\;}$	−0.37	−0.4	−0.05	−0.36	−0.04	−0.26
$T_{\mathrm{diff}\;}$	0.5	0.19	−0.08	0.21	−0.06	0.14
${\mathrm{UVENT}}_{\mathrm{roof}}$	1	1	1	1	1	1

**Table 5:** Statistical analysis of the linear and nonlinear correlation between the potential inputs and the target vent opening signal.

	$UVENT_{roof}$					
	Pearson’s Coefficient	Spearman’s Rank Coefficient	Kendall’s Rank Coefficient	Regression Analysis	Prior Knowledge about Physical Intractions	Inputs Pre-Selection
$\mathrm{CO}2_{\mathrm{out}}$	Weak correlation	No correlation	No correlation	Weak non-monotonic negative correlation	Not influenced by greenhouse vent opening. The network could implicitly consider it a reference for the difference between inside and outside climate.	Eliminated
$H_{\mathrm{out}}$	Weak negative linear correlation	Weak negative correlation when ${\mathrm{UVENT}}_{\mathrm{roof}}>0\%$	$\mathrm{Weak}\;\mathrm{negative}\;\mathrm{correlation}\;\mathrm{when}\;{\mathrm{UVENT}}_{\mathrm{roof}}>0\%$	Monotonic negative nonlinear correlation	Not influenced by greenhouse vent opening. The network could implicitly consider it a reference for the difference between inside and outside climate.	Eliminated
${\mathrm{RAD}}_{\mathrm{in}}$	Significant positive linear correlation	Moderate positive correlation when ${\mathrm{UVENT}}_{\mathrm{roof}}>0\%$	$\mathrm{Moderate}\;\mathrm{positive}\;\mathrm{correlation}\;\mathrm{when}\;{\mathrm{UVENT}}_{\mathrm{roof}}>0\%$	Monotonic positive nonlinear correlation	Not influenced by greenhouse vent opening. Considered as a reference for the diurnal and nocturnal periods, knowing that vents are mostly closed at night.	Selected
$T_{\mathrm{out}}$	Moderate positive linear correlation	Moderate positive linear correlation	Moderate positive linear correlation	Monotonic positive linear correlation	Not influenced by greenhouse vent opening. $\mathrm{Probably}\;\mathrm{correlated}\;\mathrm{because}\;\mathrm{it}\;\mathrm{increases}\;\mathrm{in}\;\mathrm{the}\;\mathrm{midday}\;\mathrm{by}\;\mathrm{solar}\;\mathrm{radiation},\;\mathrm{which}\;\mathrm{makes}\;\mathrm{its}\;\mathrm{evolution}\;\mathrm{somehow}\;\mathrm{similar}\;\mathrm{to}\;{\mathrm{UVENT}}_{\mathrm{roof}}$.	Eliminated
$W_{v}$	Weak correlation	Weak correlation when ${\mathrm{UVENT}}_{\mathrm{roof}}>0\%$	Weak correlation	Weak correlationAssociated non-monotonic variations	Not influenced by greenhouse vent opening. The network could consider its effect on the ventilation rate, in turn, on the greenhouse air variables implicitly throught vent opening.	Selected
$\mathrm{CO}2_{\mathrm{in}}$	Weak negativelinear correlation	Weak positive correlation when ${\mathrm{UVENT}}_{\mathrm{roof}}>0\%$	$\mathrm{Weak}\;\mathrm{positive}\;\mathrm{correlation}\;\mathrm{when}\;{\mathrm{UVENT}}_{\mathrm{roof}}>0\%$	Non-monotonic nonlinear correlation when ${\mathrm{UVENT}}_{\mathrm{roof}}>0\%$	Rapidly influenced by vent opening. $\mathrm{Considered}\;\mathrm{to}\;\mathrm{have}\;\mathrm{the}\;\mathrm{fastest}\;\mathrm{reaction}\;\mathrm{to}\;{\mathrm{UVENT}}_{\mathrm{roof}}$.	Selected
$H_{\mathrm{in}}$	$\mathrm{Moderate}\;\mathrm{negative}\;\mathrm{linear}\;\mathrm{correlation}\;\mathrm{when}\;{\mathrm{UVENT}}_{\mathrm{roof}}>0\%$	Moderate negative correlation when ${\mathrm{UVENT}}_{\mathrm{roof}}>0\%$	$\mathrm{Moderate}\;\mathrm{negative}\;\mathrm{correlation}\;\mathrm{when}\;{\mathrm{UVENT}}_{\mathrm{roof}}>0\%$	Negative monotonic nonlinear correlation	Rapidly influenced by vent opening. $\mathrm{Considered}\;\mathrm{to}\;\mathrm{have}\;a\;\mathrm{high}\;\mathrm{sensitivity}\;\mathrm{to}\;{\mathrm{UVENT}}_{\mathrm{roof}}$.	Selected
$T_{\mathrm{in}}$	Moderate positive linear correlation	Moderate positive correlation when ${\mathrm{UVENT}}_{\mathrm{roof}}>0\%$	$\mathrm{Weak}\;\mathrm{positive}\;\mathrm{correlation}\;\mathrm{when}\;{\mathrm{UVENT}}_{\mathrm{roof}}>0\%$	Non-monotonic nonlinear correlation	Influenced by vent opening. $\mathrm{Considered}\;\mathrm{to}\;\mathrm{have}\;a\;\mathrm{high}\;\mathrm{sensitivity}\;\mathrm{to}\;{\mathrm{UVENT}}_{\mathrm{roof}}$.	Selected
$\mathrm{CO}2_{\mathrm{diff}\;}$	Moderate negative linear correlation	Weak negative correlation when ${\mathrm{UVENT}}_{\mathrm{roof}}>0\%$	$\mathrm{Weak}\;\mathrm{negative}\;\mathrm{correlation}\;\mathrm{when}\;{\mathrm{UVENT}}_{\mathrm{roof}}>0\%$	Non-monotonic nonlinear correlation	Rapidly influenced by vent opening. $\mathrm{Considered}\;\mathrm{to}\;\mathrm{have}\;a\;\mathrm{high}\;\mathrm{sensitivity}\;\mathrm{to}\;{\mathrm{UVENT}}_{\mathrm{roof}}$.	Selected
$H_{\mathrm{diff}\;}$	Moderate negative linear correlation	Moderate negative correlation when ${\mathrm{UVENT}}_{\mathrm{roof}}>0\%$	$\mathrm{Weak}\;\mathrm{negative}\;\mathrm{correlation}\;\mathrm{when}\;{\mathrm{UVENT}}_{\mathrm{roof}}>0\%$	Monotonic nonlinear correlation	Rapidly influenced by vent opening. $\mathrm{Considered}\;\mathrm{to}\;\mathrm{have}\;a\;\mathrm{high}\;\mathrm{sensitivity}\;\mathrm{to}\;{\mathrm{UVENT}}_{\mathrm{roof}}$.	Selected
$T_{\mathrm{diff}\;}$	Moderate positive linear correlation	Weak positive correlation when ${\mathrm{UVENT}}_{\mathrm{roof}}>0\%$	$\mathrm{Weak}\;\mathrm{positive}\;\mathrm{correlation}\;\mathrm{when}\;{\mathrm{UVENT}}_{\mathrm{roof}}>0\%$	Non-monotonic nonlinear correlation	Influenced by vent opening. $\mathrm{Considered}\;\mathrm{to}\;\mathrm{have}\;a\;\mathrm{high}\;\mathrm{sensitivity}\;\mathrm{to}\;{\mathrm{UVENT}}_{\mathrm{roof}}$.	Selected

**Table 6:** Statistical evaluation of the obtained LSTM-based networks using different cost functions.

	All the Available Inputs				Eliminating Only $W_{v}$				Eliminating $T_{out}$, $H_{out}$ and $CO2_{out}$			
	$R^{2}$	RMSE (%)	MAE (%)	MaxAE (%)	$R^{2}$	RMSE (%)	MAE (%)	MaxAE (%)	$R^{2}$	RMSE (%)	MAE (%)	MaxAE (%)
Architecture A (LSTM-ANN)	0.77	9.8	3.94	77.66	0.74	10.04	4.04	99.03	0.77	9.64	5.33	65.76
Architecture B (LSTM-RNN)	0.61	12.76	5.98	93.05	0.75	10.32	5.43	63.42	0.8	9.13	4.45	62.94

## Data Availability

The data presented in this study are available on request from the corresponding author.
